# The paradox of argument strength: how weak arguments undermine the persuasive effects of strong arguments

**DOI:** 10.1038/s41598-024-73348-1

**Published:** 2024-09-27

**Authors:** Magdalena Obermaier, Thomas Koch

**Affiliations:** 1 Department of Media and Communication, LMU Munich, Oettingenstr. 67, 80538 Munich, Germany; 2 Department of Communication, Johannes Gutenberg-University Mainz, Mainz, Germany

**Keywords:** Persuasive communication, Strong and weak arguments, Argument strength, Adding, Averaging, Environmental social sciences, Psychology and behaviour, Public health

## Abstract

This paper analyzes effects of the mutual presentation of weak and strong arguments. Departing from the prevalent “the-more-the-better” heuristic, our research scrutinizes whether the inclusion of weak arguments enhances or diminishes the persuasive impact of strong arguments. Leveraging insights from judgment formation literature, we conducted four experimental studies on political and health-related topics to unravel whether the presenting weak arguments strengthens the persuasive effect of a strong argument (adding) or actually weakens this persuasive effect (averaging). The results show that providing supporting arguments of moderate strength along with a strong argument increases persuasion, representing an additive pattern. However, presenting weak supporting arguments along with a strong argument reduces the persuasive effect of the strong argument, representing an averaging pattern. Exposure to weak arguments diminishes the strength of strong ones, suggesting the omission of weak arguments. These findings underscore the vital role of strategically selecting arguments to optimize persuasion across disciplines.

## Introduction

Imagine you want to convince people to do something for their health (e.g., getting a certain vaccine, exercising more often, or getting regular colonoscopies), to practice more sustainable behavior in their everyday lives (e.g., reducing plastic waste, buying fair fashion, or biking to work), or to support a political agenda (e.g., voting for a certain political candidate or advocating a certain political project). For each of these areas, one can immediately think of different arguments to convince others. However, only some of these arguments may seem strong (e.g., exercising more often will decrease the risk of chronic disease and is good for bones and muscles), whereas others may seem weaker (e.g., exercising more often can be fun and can be a reason to buy fancy activewear).

After you have gathered different arguments, you would probably think about how to present them to convince others of your position. For instance, you might reflect upon whether to persuade people by presenting the weak or the strong arguments first, which classically has been studied as the persuasive effects of the climax or anticlimax order of arguments^1,2^. However, before thinking about how to arrange the strong and weak arguments, you need to ask yourself whether it is at all helpful to present the weak arguments along with the strong ones.

The research conducted so far has largely overlooked this fundamental question. Although numerous studies have examined the effects of one- and two-sided argumentation and the impact of climax and anticlimax order in argumentative speech, research has long neglected the question of how the persuasive effects of strong and weak arguments interact^3–7^. The present study addresses a necessary prior step by analyzing how displaying weak arguments can affect the persuasive effects of strong arguments. We draw on studies on impression formation and consumers’ evaluation of product bundles (i.e. several products that are sold together as a package)^8,9^. This research suggests that displaying both strong and weak arguments could lead to either increased persuasion, if the recipients mentally add the weak arguments to the strong ones, or decreased persuasion, if the recipients form a mental average of the weak and strong arguments^10^. The present study examines which of these patterns emerges if both weak and strong arguments are presented^7,11^. In this investigation, online experiments explored interdisciplinary subjects to assess the impact of combining weak or moderate arguments with a strong argument.

### Adding and averaging

Research on impression formation and consumers’ evaluation of product bundles suggests that there are two main ways to process mildly favorable information when it is presented alongside highly favorable information: an adding and an averaging pattern^10,12^. This section delineates the characteristics of these patterns and their application in processing both weak and strong arguments. Arguments are defined as reasons given to support or deny something^13^. The single most used argument type in practice and research are pragmatic arguments or, as synonymously called in normative argumentation theory, arguments from consequences^14,15^. These pragmatic arguments or arguments from consequences respectively are defined as “a species of practical reasoning where a contemplated policy or course of action is positively supported by citing the good consequences of it”^16^.

If people are presented with weak and strong arguments in support of an issue, they may implicitly weight and mentally add the arguments to form an attitude (*adding*). If this processing occurs, the mutual presentation of weak and strong arguments should lead to more favorable attitudes regarding the issue^17,18^. This pattern assumes that individuals evaluate each argument as independent entities. In line with this idea, this mode has been labeled as “piecemeal” or “analytic” processing^12^. This processing style has been found in impression formation and presenters’ evaluation of product bundles^10,17,19^. Research on the effects of one-sided argumentation has implicitly assumed adding, relying on a “the-more-the-better” heuristic^20^. Given this mechanism occurs, people will be more convinced after the mutual presentation of weak and strong arguments.

However, when individuals are confronted with strong and weak arguments in favor of an issue, they can also *average* the different arguments. There are two ways how this averaging may work^21^: According to algebraic averaging models, individuals adjust the weight they ascribe to each attribute of a person or product—or each argument—in accordance with the weights assigned to the other attributes or arguments. Hence, weighting the weak arguments can weaken the persuasive effect of the strong arguments^22^. Individuals could also judge each piece of information (or argument) separately in terms of its weight (or strength) and then average these separate judgments^23^. Because, in both cases, individuals are looking at the big picture and judging each piece of information in relation to all the information provided, this processing is also referred to as “holistic”^24^. This has been studied under the label of a dilution effect in impression formation^25,26^. Assuming that this pattern occurs in persuasive communication, individuals will not be as convinced after the joint presentation of weak and strong arguments as they would be after the presentation of a strong argument alone.

### Effects of the mutual presentation of weak and strong arguments

Adding and averaging patterns have been mostly demonstrated in impression formation or product bundle evaluation, with participants evaluating moderately and highly favorable features^10^. It is not clear whether these effects also occur in persuasive communication when both weak and strong arguments are presented.

Individuals evaluating a person or product assume that its traits or features are not independent of one another because all of these characteristics make up a personality or product quality. It is thus not surprising that averaging-like processes occur in the evaluation of people and products^9^. However, will people perceive a persuasive speech, intended to convince recipients to support a political agenda or to do something for their health, for instance, as a unified and coherent entity or will they perceive the different (weak and strong) arguments as independent entities?

There is only few evidence so far that recipients’ attitudes might deteriorate if strong and weak arguments are presented^10,11^. Hence, in this study we were interested in whether the presentation of weak arguments in addition to a strong argument reduces or enhances the persuasive effects of the strong argument.

## Overview: the present research

In four consecutive experiments, we varied topics, argument numbers, and strengths across studies. Study 1a examined the influence of presenting one strong and three weak arguments favoring sustainability-focused renovation, while Study 1b replicated this for railroad construction. Study 2 investigated persuasive effects by jointly presenting one strong and four weak arguments for sustainability-driven urban swimming pool renovation, compared to a single strong argument. Study 3 explored impacts of presenting one strong argument with three weak or moderately strong arguments advocating for a health check-up, contrasting with a single strong argument. Detailed designs and findings for each study follow.

## Study 1a: investigating the mechanisms

Previous findings suggest that the presentation of mildly favorable information can dilute the persuasive effect of favorable information, resulting in an averaging pattern^27^. Research also shows that mildly favorable information can be added to highly favorable information, resulting in a cumulative judgment^10,28^. The first study explores whether the mutual presentation of weak and a strong argument will result in averaging or adding.

### Results

Univariate ANOVA showed that participants were most supportive of the renovation when they saw both the three weak arguments and the strong argument (*n* = 50, *M* = 4.22, *SD* = 0.71), compared with the presentation of the strong argument alone (*n* = 50, *M* = 3.88, *SD* = 0.79), or only the three weak arguments (*n* = 53, *M* = 3.49, *SD* = 0.93), *F* (2, 150) = 10.47, *p* < .001, η²_part_ = 0.12 (one-tailed). Duncan post hoc tests revealed significant differences between all three groups (*p* < .05).

### Discussion

Given these results, our findings in Study 1a indicate adding: Providing three weak arguments in favor of renovating the cultural center supported the persuasive effect of the strong argument in favor, compared with providing the strong argument alone.

### Method

*Participants*. The sample comprised 156 participants (53.6% female; age: *M* = 48.05 years, *SD* = 15.92) who were recruited using an online access panel^30^. Three participants had to be excluded due to missing data. Participation was voluntary and unpaid. Informed consent was obtained from all subjects and the experiment was performed in accordance with relevant guidelines and regulations. Our study did not require IRB approval because it used low-risk stimuli while adhering to key principles ensuring participant safety and well-being: voluntary participation with informed consent, complete anonymity, the right to withdraw at any time, and thorough debriefing. Besides that, IRB consent is not obligatory by local legislation.

*Design and Procedure*. We used a 3 × 1 between-subjects design, varying whether participants received (I) three weak arguments; (II) one strong argument; or (III) the strong argument and the three weak ones, with all arguments in favor of the renovation of a cultural center. Participants were randomly assigned to one of the three conditions.

Participants read a fictitious Facebook post from a local politician (“Kurt Oberhofer”) who argued in favor of the renovation of a cultural center in his city (Tables 1 and 2). We selected arguments most common in practice and research supporting a policy or project by mentioning the beneficial outcomes^14,16^. Applying a perspective related to norms of assessment of argument quality, the arguments are characterized by the criterium of desirability^14,16,29^: while the strong argument is very desirable (environmental protection through renovation), the weak arguments score lower in desirability (more comfortable use of the center through renovation). In the first condition, three weak arguments were employed, mentioning the installation of additional windows, the addition of indirect foyer lighting, and the replacement of broken terrace tiles. The second condition featured a single strong argument, emphasizing the replacement of old heating with an environmentally friendly system and insulation of walls to substantially reduce energy demand and air pollution. In the third condition, the politician presented both the three weak arguments and the strong one, with the strong argument advocating for the highly desirable outcome (environmental protection), while the weak arguments focused on the less desirable aspects (enhanced comfort)^29^.

**Table 1:** Stimulus materials.

	Arguments		
	Strong		Weak
*Study 1a*	The Cultural Center’s building insulation and heating system are very outdated. In the course of the renovation, the old heating system will be replaced with a more environmentally friendly system, and the walls will be insulated, which will significantly reduce energy demand and thus air pollution.		The large hall will get another window to make it brighter. In addition, to make the foyer look friendlier, indirect lighting will be installed on the ceiling. Furthermore, broken terrace tiles will be replaced, which should have been done years ago.
	*Strong*		*Weak*
*Study 1b*	With the new track layout, the regional train will only take about one hour instead of one hour and 30 min. For passengers, this means a time savings of around 30 min.		Another positive aspect for passengers is that the route will be less curvy. The regional train will be able to run somewhat more quietly on the new tracks. Finally, the higher track bed ensures less vibration during the journey.
	*Strong*		*Weak*
*Study 2*	The refurbishment will replace the heating system with an environmentally friendly system that will significantly reduce air pollution and energy consumption.		The lighting technology will be brought up to date. As part of the refurbishment, scratches on tiles in the bath and shower area will be removed. As a further measure, the changing area is to be extended by two new individual cubicles. In addition, a new entrance area, which will be covered by a roof, will be built.
	*Strong*	*Moderate strength*	*Weak*
*Study 3*	A regular full-body health check can detect cardiovascular diseases and metabolic disorders at an early stage.	The full-body health check will analyze the extent of emotional stress factors. In the long term, the tests will reduce the financial burden on the healthcare system because illnesses can be detected and treated at an early stage. On request, participants will also receive tips on changing their diets after the full-body health check.	All participants will receive the video “Lowering cholesterol without pills” free of charge. Completing the full-body health check will give participants insight into everyday life in a hospital. The full-body health check will offer the opportunity to get out of one’s daily routine.

*Notes*. This is the English translation of stimulus materials that were originally written in German.

**Table 2:** Full filler text of stimulus materials.

***Study 1b***	**Renovation project: Kulturwerkstatt Neustadt** Dear citizens, At the beginning of next week, the city council will be discussing the renovation of the Kulturwerkstatt Neustadt. In order for such a measure to be implemented, we need a majority in the city council. I am in favor of a comprehensive renovation, even if it is costly. I would like to explain my position here. *Why should the Kulturwerkstatt Neustadt be renovated?* [ARGUMENT(S) ABOUT HERE] We will vote next week on whether the renovation project should be implemented. I will do all I can to support the renovation. In the event of a positive decision, the next step would be a referendum. In this case, I appeal to you: get involved! Vote for the renovation of the Kulturwerkstatt. *Kurt Oberhofer, Member of the City Council*
***Study 1b***	**Decision on route expansion? Interview with Regional Council member Gerd Schultz about the Wesel line extension** *By Svenja Fichter* Düsseldorf. At the beginning of next week, the regional council of the district government will be discussing a possible extension of the Wesel railroad line. We spoke to a member of the regional council, Gerd Schultz, about the background to the expansion and the next steps. *Mr. Schultz*,* according to our information*,* the regional council has a first draft for the expansion of the railroad line in the Wesel area. Why now?* The city of Wesel approached us some time ago with this proposal for the expansion of the line. In order for the plans to be implemented, the regional council must first discuss whether state funding for the project is possible. However, as the plenary session does not meet every week, the earliest possible date is the beginning of next week. *What exactly does the construction proposal envisage?* In principle, it is about redesigning the route of the regional line in both directions. This involves a route of 43 km, which is to run further north-east from its old position. We will be discussing this in plenary. *Why should the route be upgraded?* [ARGUMENT(S) ABOUT HERE] *What are the next steps after next week’s meeting?* We will vote soon on whether the construction project should be implemented. In the event of a positive decision, the next step would be a referendum.
***Study 2***	**Municipal utilities present restructuring plan at municipal council meeting: Waidhofen indoor swimming pool to be renovated** *By Lea Meissner* Marlch-Waidhofen. The renovation of Waidhofen’s indoor swimming pool was on the agenda of last Wednesday’s municipal council meeting. The majority of the town representatives voted in favor of the renovation with a decision in principle. A final decision will be made by the residents in a referendum in December. *Measures* Stadtwerke Managing Director Kerin Winkel presented the conversion plans at the meeting. The preliminary concept includes the following measures: [ARGUMENT(S) ABOUT HERE] *Next steps* “We recommend carrying out the work in a single construction phase from June to September 2019,” said Managing Director Winkel. The pool could remain open as normal until the start of construction. The estimated costs for the refurbishment currently amount to 240,000 euros. Mayor Peter Walch commented on the financing as follows: “We are expecting a proportionate subsidy from the state. The corresponding application has already been submitted.” The total costs to be incurred by the municipality will be determined before the referendum. On December 8, residents will then have the opportunity to cast their vote for or against the renovation.
***Study 3***	At Privatklinikgruppe Dr. Schneider AG, we also offer an annual full-body health check especially for young adults. There are numerous reasons to have an annual full-body health check. *Why should you have a full-body health check?* [ARGUMENT(S) ABOUT HERE] If you have opted for our complete health check, please register by telephone or e-mail approximately two weeks before your desired appointment. If you wish, you can also ask our doctors for a non-binding consultation in advance. *Kurt Oberhofer*,* Press Spokesman of the Private Hospital Group Dr. Schneider AG*

*Notes*. This is the English translation of stimulus materials that were originally written in German.

*Measures*. We measured participants’ attitudes towards renovating the cultural center using a 5-point semantic differential scale of five polar adjectives in response to “I believe that a renovation of the cultural center is…”. The bipolar adjective pairs were “not necessary – necessary,” “not useful – useful,” “not reasonable – reasonable,” “bad – good,” and “negative – positive” (α = 0.93, *M* = 3.85, *SD* = 0.87). We opted for odd-numbered semantic differential scales common in research on persuasive communication in the following to ensure the greatest possible comparability. Also, we wanted to allow participants to place themselves in the middle of the scale to express an ambivalent attitude.

Lastly, participants were asked to rate the perceived strength of each presented argument on a 5-point semantic differential scale ranging from “weak” to “strong.”

*Treatment check*. A repeated measure ANOVA (*n* = 45 in each group) showed that the strong argument (achieving a better energy balance) was rated significant stronger (*M* = 4.22, *SD* = 0.97) than any of the other three arguments (installing of additional windows: *M* = 3.13, *SD* = 1.39; adding indirect foyer lighting: *M* = 3.16, *SD* = 1.33; replacing terrace tiles: *M* = 3.33, *SD* = 1.38), *F* (3, 132) = 12.83, *p* < .001, η²_part_ = 0.23 (one-tailed). Duncan post hoc tests revealed significant differences (*p* < .05) between the strong and each of the weak arguments, indicating that the manipulation was successful.

## Study 1b: investigating the mechanisms

The adding pattern found in Study 1a contradicts the few previous studies that have examined effects of the mutual presentation of weak and strong arguments^7^. To rule out that this finding is restricted to the specific topic and the arguments used in Study 1a, we replicated the experiment using a different setting.

### Results

Participants who saw only the strong argument supporting the railroad construction had a more positive attitude toward the project (*n* = 57, *M* = 3.75, *SD* = 0.90) than did those who were provided with three weak arguments and the strong argument (*n* = 58, *M* = 3.47, *SD* = 0.79) or those receiving three weak arguments alone (*n* = 57, *M* = 2.70, *SD* = 0.94), *F* (2, 169) = 21.78, *p* < .001, η²_part_ = 0.21 (one-tailed). Duncan post hoc tests revealed significant differences between all three groups (*p* < .05). Thus, the presentation of three weak arguments reduced the persuasive effects of a standalone strong argument, indicating an averaging. This finding is in contrast with the results of Study 1a.

### Discussion

Whereas Study 1a revealed an adding pattern, Study 1b showed an averaging pattern. We believe that participants perceived the strength of the (supposedly) weak arguments in Study 1a as too similar to that of the designated strong argument: The mean value of the strength of the strong argument was 3.92, the strength of the weak arguments was rated 3.14, resulting in a total difference of 0.78 in Study 1a. In Study 1b, the mean value of the strength of the strong argument was 4.13, the strength of the weak arguments was rated 2.82, resulting in a total difference of 1.31. The discrepancy between the two differences could have triggered different patterns.

### Method

*Participants*. A sample of 173 participants (43.9% female; age: *M* = 37.78 years, *SD* = 14.98) was recruited using an online access panel^30^. One participant had to be excluded from further analysis due to missing data. Participation was voluntary and unpaid. Informed consent was obtained from all subjects and the experiment was performed in accordance with relevant guidelines and regulations. IRB approval was not necessary for this study as well, which is in accordance with local legislation.

*Design and Procedure*. We again used a 3 × 1 between-subjects design, providing participants either with (I) three weak arguments; (II) one strong argument; or (III) the strong argument and the three weak ones, with all arguments supporting a railroad construction project. Participants were randomly assigned to one of the three conditions.

Participants read a fictitious article published in a national newspaper. In this article, a local politician (“Gerd Schultz”) was interviewed about a railroad construction project and argued in favor of the project planned for his region (Tables 1 and 2). In the first condition, he presents three weak arguments, namely that after the construction work the railroad would be less curvy, there would be less vibration, and the ride would be quieter. In the second condition, he made the strong argument that because of a new rail track, passengers would save 30 min on the ride. In the third condition, he presented both the three weak arguments and the strong one. Thereby, the strong argument advocating the construction project (time saving) was more desirable for passengers than the weak arguments (more pleasant driving experience)^14^.

*Measures*. As dependent variable, we measured attitudes toward the railroad construction using a 5-point semantic differential scale of five polar adjectives in response to “I believe that the railroad construction is…”. The bipolar adjective pairs were the same as in the first study: “not necessary – necessary,” “not useful – useful,” “not reasonable – reasonable,” “bad – good,” and “negative – positive” (α = 0.92, *M* = 3.31, *SD* = 0.98).

Again, lastly participants were asked to assess the perceived strength of each presented argument on a 5-point semantic differential scale ranging from “weak” to “strong.”

*Treatment check*. A repeated measure ANOVA (*n* = 55 in each group) showed that the strong argument (time saving of 30 min) was clearly considered to be stronger (*M* = 4.13, *SD* = 0.86) than any of the three weak arguments (track less curvy: *M* = 2.49, *SD* = 1.29; less vibration: *M* = 2.78, *SD* = 1.26; quieter ride: *M* = 3.20, *SD* = 1.13), *F* (3, 162) = 51.71, *p* < .001, η²_part_ = 0.49 (one-tailed). Duncan post hoc tests revealed significant differences (*p* < .05) between the strong and each of the weak arguments, indicating that the manipulation was successful.

## Study 2: replicating the averaging effect

Based on our findings in Studies 1a and 1b, we suggest that averaging occurs only when participants are presented with a strong argument alongside clearly weaker ones. In Study 1a, weak arguments had a mean of 3.21 (*SD* = 1.37) and strong arguments had a mean of 4.22 (*SD* = 0.97), yielding a Cohen’s *d* of -0.85. In Study 1b, the weak arguments had a mean of 2.82 (*SD* = 1.23) and strong arguments a mean of 4.13 (*SD* = 0.86), with a Cohen’s *d* of -1.23. We hypothesize: The presentation of very weak arguments along with a strong argument evokes an averaging pattern (*H1*).

To assess this assumption, we aimed to replicate the averaging effect observed in Study 1b using a different topic, ensuring a substantial strength disparity between weak and strong arguments. Perceived argument strength was pretested. We hypothesized that individuals exposed to four distinctly weak (yet non-specious) arguments alongside one strong argument supporting a public swimming pool renovation would hold less favorable opinions of the project compared to those presented with only the strong argument.

### Results

Study 2 showed that participants who saw the standalone strong argument in favor of the renovation of the public swimming pool were more positive about the project (*n* = 62, *M* = 4.32, *SD* = 0.64) than those who were presented with four weak arguments along with the strong argument (*n* = 56, *M* = 4.04, *SD* = 0.77), *t* (116) = 2.11, *p* = .02, *d* = 0.39 (one-tailed). Thus, *H1* was supported.

### Discussion

The findings of Study 2 confirm our assumption of an averaging in the joint presentation of a strong argument along with clearly weak arguments and, hence, replicate the findings of Study 1b. Thus, presenting arguments that were clearly weak thus reduces the persuasive effect of the strong argument. Conversely, in Study 1a, the joint presentation of moderately strong arguments with a strong argument enhanced the persuasive effect of the latter, indicating adding. These findings suggest that the perceived strength of weak arguments is crucial in determining which pattern is at play.

### Method

*Pretest*. In a pretest with students attending a university lecture (*n* = 30, 73.3% female; age: *M* = 25.93 years, *SD* = 3.07), we identified strong and weak arguments in favor of a renovation project for a public swimming pool. Participation was voluntary and unpaid. Informed consent was obtained from all subjects and the pretest was performed in accordance with relevant guidelines and regulations. Participants were asked to assess 16 different arguments in favor of the renovation project on 5-point semantic differential scales ranging from “weak” to “strong.” We selected the four arguments that were perceived as weakest (adding single cabins in the changing room: *M* = 2.13 *SD* = 1.04; removing scratches on the tiles: *M* = 2.23, *SD* = 1.07; renovating the lighting: *M* = 2.30, *SD* = 0.88; adding a roof over the entrance area: *M* = 2.43, *SD* = 0.82) and one argument that was perceived as strong (reducing energy consumption: *M* = 4.43, *SD* = 0.68). The substantial difference between the weak and strong arguments resulted in a large effect size (Cohen’s *d* = -2.62), indicating a clear and pronounced distinction in how participants evaluated the strength of these arguments. The distinguishing feature of the arguments lies in the desirability of the outcomes, with the strong argument favoring environmental protection, while the weak arguments prioritize enhanced comfort. The selected arguments are characterized – in line with considerations related to norms of assessment of argument quality – by the fact that the strong argument (environmental protection) is more desirable than the weak arguments (more comfortable use of the swimming pool).

*Participants*. We recruited a sample of 118 participants via social media and university classes (67.8% female; age: *M* = 29.04 years, *SD* = 10.66). Participation was voluntary and unpaid. Informed consent was obtained from all subjects and the experiment was performed in accordance with relevant guidelines and regulations. IRB approval was not necessary for this study as well, which is in accordance with local legislation.

*Design and Procedure*. In a 2 × 1 between-subjects design, we varied the presentation of (I) one strong argument or (II) one strong argument and four weak ones, with all arguments in favor of an urban swimming pool renovation project. Participants were randomly assigned to one of the two conditions.

We asked subjects to read a fictitious online article published in a local newspaper. In this article, a local politician (“Kerstin Winkel”) was cited supporting a public swimming pool renovation (Tables 1 and 2). In the initial condition, she presented a single strong argument, emphasizing the installation of an environmentally friendly heating system to significantly reduce air pollution and energy consumption. In the second condition, she reiterated the strong argument and expanded by mentioning the addition of two single cabins in the changing room, the removal of scratches from shower tiles, renovation of lighting equipment, and construction of a roof over the entrance.

*Measures*. We measured attitudes toward the public swimming pool renovation project using a 5-point semantic differential scale of five polar adjectives in response to “I believe that a renovation of the swimming pool is…”. The bipolar adjective pairs were the same as in Study 1: “not necessary – necessary,” “not useful – useful,” “not reasonable – reasonable,” “bad – good,” and “negative – positive” (α = 0.85, *M* = 4.19, *SD* = 0.71).

## Study 3: replicating adding and averaging

Study 1b and Study 2 showed that the joint presentation of significantly weak arguments and a strong argument can reduce the persuasive effect of the strong argument (averaging). Study 1a demonstrated that the presentation of arguments perceived as moderately strong along with a strong argument seems to increase the persuasive effect of the strong argument (adding). Nuances in the perceived strength of the weak arguments may determine which mechanism occurs.

Therefore, we hypothesize: The presentation of very weak arguments along with a strong argument evokes an averaging mechanism (*H2a)*. And: The presentation of moderately strong arguments along with a strong argument leads to an adding mechanism (*H2b*). To test these assumptions, we conducted another online experiment preceded by a pretest.

### Results

Study 3 demonstrated that the participants had more positive attitudes toward the full-body health check when only the strong argument was presented (*n* = 45, *M* = 3.56, *SD* = 0.96), compared with the presentation of the three clearly weak arguments along with the strong argument (*n* = 42, *M* = 3.11, *SD* = 1.05). Therefore, *H2a* was supported. However, support for the full-body health check was highest among the young adults who were presented with the three arguments of moderate strength along with the strong argument (*n* = 36, *M* = 4.07, *SD* = 0.91), *F* (2, 120) = 9.33, *p* = .001, η²_part_ = 0.14 (one-tailed), which supports *H2b*. Duncan post hoc tests revealed significant differences between all three groups (*p* < .05).

### Discussion

As assumed, both an adding and an averaging pattern emerged in Study 3. The presentation of three clearly weak arguments reduced the persuasive effect of the strong argument supporting a full-body health check, which corresponds to an averaging pattern. Conversely, providing young adults with three arguments of moderate strength in favor of the health check enhanced the persuasive effect of the strong argument, reflecting an adding pattern. Interestingly, the very low scores for the weak arguments (*M* = 1.82, 1.47, 1.45) suggest that these arguments might be perceived as irrelevant. Hoeken et al. define irrelevant arguments as those that do not impact the target audience’s decision-making process or distinguish the product from its competitors^14^. When arguments are perceived as irrelevant, they likely fail to contribute meaningfully to the persuasion process, thereby reducing the effectiveness of the strong argument. These findings show that the perceived strength of the weak arguments is decisive in determining whether averaging or adding occurs when these arguments are presented in combination with a strong argument.

### Method

*Pretest*. We recruited a sample of students at a particular university (*n* = 55, 80% female; age: *M* = 22.50 years, *SD* = 5.11) and asked them to assess the strength of 21 arguments supporting the idea of a full-body health check for young adults up to 35 years on 5-point semantic differential scales ranging from “weak” to “strong.” Informed consent was obtained from all subjects and the pretest was performed in accordance with relevant guidelines and regulations. Based on their assessments, we chose the three weakest arguments (*M* < 2.00; free video on lowering cholesterol: *M* = 1.82, *SD* = 0.98; hospital insights: *M* = 1.47, *SD* = 0.72; breaking daily routine: *M* = 1.45, *SD* = 0.74), three arguments of moderate strength (3.00 < *M* < 3.50: dietary tips: *M* = 3.16, *SD* = 1.14; recognizing emotional stress: *M* = 3.42, *SD* = 1.03; financial relief for the healthcare system: *M* = 3.47, *SD* = 1.23), and the strongest argument (early detection of cardiovascular diseases: *M* = 4.55, *SD* = 0.81) for the full-body health check. The large difference between the weak and strong arguments yielded a substantial effect size (Cohen’s *d* = -3.67), while the expected difference between moderately strong and strong arguments produced a more moderate effect size (Cohen’s *d* = -1.22).

*Participants*. A total of 123 young adults (18–35 years old) were recruited mainly via e-mail distribution lists of lectures at two particular universities (78.9% female; age: *M* = 23.79 years, *SD* = 4.54). Participation was voluntary and unpaid. Informed consent was obtained from all subjects and the experiment was performed in accordance with relevant guidelines and regulations. IRB approval was not necessary for this study, too, which is in accordance with local legislation.

*Design and Procedure.* In a 3 × 1 between-subjects design, we varied whether participants were presented with (I) a strong argument; (II) the strong argument along with three clearly weak arguments; or (III) the strong argument along with three moderately strong arguments. Participants were randomly assigned to one of the conditions.

Subjects were asked to read a fictitious Facebook post from a private hospital group in which the hospital group’s spokesperson (“Kurt Oberhofer”) promoted an annual full-body health check for young adults (Tables 1 and 2). In the initial condition, he presented three clearly weak arguments, including the offer of a free video on lowering cholesterol, insights into hospital procedures, and a chance to break the daily routine. These were coupled with the strong argument emphasizing the early detection of cardiovascular diseases through the full-body health check. In the second condition, he offered three arguments of moderate strength, highlighting dietary tips, recognition of emotional stress factors, and long-term financial relief to the healthcare system due to early disease detection and treatment. These were presented alongside the strong argument. In the third condition, only the strong argument was presented. Again, it becomes clear that the chosen strong argument for the health check has more desirable consequences for patients (early detection of disease) than the moderate arguments (e.g., receiving tips for health promotion) and the very weak arguments (e.g., receiving health-related freebies).

*Measures*. We measured attitudes toward the full-body health check using a 5-point semantic differential scale of five polar adjectives in response to “I believe that the full-body health check is…”. The bipolar adjective pairs were the same as in the first studies: “not necessary – necessary,” “not useful – useful,” “not reasonable – reasonable,” “bad – good,” and “negative – positive” (α = 0.94, *M* = 3.56, *SD* = 1.04).

## General discussion

The present study examined the persuasive impact of jointly presenting weak and strong arguments, an aspect overlooked in persuasive communication research, which typically focused on one- and two-sided argumentation, order effects, and argument quality assessments^3,31,32^. Drawing on insights from research on impression formation and product bundles, two processing styles for weak and strong arguments were identified. The first, an adding pattern, involves individuals treating weak and strong arguments as separate entities (piecemeal processing), enhancing the persuasive effects of the latter^9^. The second, an averaging pattern, sees weak arguments diminishing the persuasive impact of strong arguments as individuals form an overall impression of the presented arguments (holistic processing)^12^.

In a series of four consecutive experiments covering interdisciplinary political and health-related topics, our findings consistently demonstrate that the inclusion of clearly weak arguments diminishes the persuasive impact of a strong argument. This effect was observed across studies examining attitudes towards supporting railroad construction (Study 1b), renovation (Study 2), and a full-body health check (Study 3). Hence, consistent with prior research on impression formation and product evaluation, the averaging pattern manifests when clearly weak and strong arguments are jointly presented^7,10^. Additionally, our experiments reveal that arguments of moderate strength enhance the persuasive impact of a concurrently presented strong argument (Study 1a, Study 3). Thus, the intended effect of offering various arguments of different strength (“more is better”) only occurs if arguments are presented that are at least of moderate strength^17^. Thus, only provided that is the case, a “the-more-the-better” heuristic seems appropriate.

These findings hold significant implications across disciplines and for practitioners, offering avenues for future research. The field of persuasion research should consider these findings and derive recommendations regarding the arrangement of strong and weak arguments. A crucial distinction must be made between clearly weak arguments, which hinder persuasive effects, and moderately strong arguments, which amplify the persuasiveness of strong arguments. Our results suggest that only the joint presentation of very weak arguments with a strong argument—following the specific order used in our experiments—can reduce the persuasive effects of the strong argument. This insight emphasizes that clearly weak arguments can counterproductively diminish the persuasive impact of strong arguments, but this effect is context-dependent and related to the particular sequence of argument presentation used in our study. Future research should explore how varying the order of argument presentation might affect these outcomes.

The results carry relevance for diverse domains, including political, health, science, environmental, and advertising communications. Tailoring argument selection to the audience is as paramount as ever, as the perceived strength of an argument varies among individuals. For example, convincing someone to quit smoking may involve presenting the strong argument of preventing lung cancer at an older age, though its effectiveness depends on individual perspectives. Young people, prioritizing the search for a partner, may find the supposedly weaker argument of improved breath smell more compelling than older individuals in long-term marriages. Future research should address these nuanced perceptions of argument strength.

The findings are limited in several ways. First, in all studies we presented one strong and three to four weak arguments. As little is known regarding the effects of the mutual presentation of weak and strong arguments, it made the most sense to work with one argument that is clearly perceived as strong and several arguments that are perceived as weak or moderately strong, which we ensured using manipulation checks and pretests. Nevertheless, we enhanced the robustness of our findings by employing diverse topics and communicators. In Study 3, we labeled arguments as “moderate” when their mean strength was perceived around the scale midpoint, while those well below (above) were categorized as weak (strong). Future research should aim to replicate our results with a broader range of strong, weak, and moderately strong arguments, systematically manipulating their quantity and quality.

We selected arguments differing in the desirability of their positive outcomes^33^. Future research should introduce clearly specious arguments alongside weak ones to compare their effects and mechanisms^7^. It is conceivable that an averaging pattern may emerge with flimsy arguments, because persuasion knowledge and reactance are triggered, which, in turn, reduce the persuasive effects^19,34^.

Moreover, to ensure consistency across the studies, we maintained the same order of argument presentation in all our experiments. Specifically, we consistently presented the weak arguments first, in an identical sequence, followed by the strong argument. This methodological choice aimed to eliminate any potential confounding variables associated with argument order between the four studies. However, we recognize that the order in which arguments are presented can influence the persuasive outcomes. Presenting weak arguments before a strong argument can create a contrast effect, where the strong argument appears significantly more persuasive in comparison to the preceding weak arguments. This occurs because the strong argument is evaluated relative to the weaker points that came before it, making it seem more compelling by contrast. The contrast effect can lead to inflated perceptions of the strong argument’s effectiveness, as the stark improvement from weak to strong may enhance its perceived impact. Future research should explore how varying the sequence of weak and strong arguments might impact the results. Investigating whether the averaging effects observed are contingent on the specific order of argument presentation would provide valuable insights into the underlying cognitive processes involved in persuasion.

Second, to minimize pre-existing attitudes towards the positions we sought to convince subjects of, we deliberately selected low-involvement settings. We focused on topics unfamiliar to participants, thus having lower relevance to them, such as specific projects or a health check in certain cities. Replicating these experiments with more important issues to participants, like climate change or vaccines, would be valuable. In high-involvement settings, the evaluation of weak arguments may vary; highly involved participants might consider objectively weak arguments to be relatively strong (resulting in adding), while low-involved participants may perceive these weak arguments as particularly weak (resulting in averaging)^35^. Along these lines, future research should replicate these studies while systematically varying communicator characteristics such as credibility and explicitly considering pre-existing attitudes on part of the participants (e.g., their level of generalized political trust). For this purpose, future studies could explore a broader range of topics, including those where participants have ideological stakes or pre-existing attitudes, as well as more neutral topics with minimal belief implications.

A further limitation is that Study 2 relied on a student sample to assess argument strength, which may result in a higher perceived strength of arguments compared to a more diverse general population. Consequently, while the observed patterns regarding how weak and strong arguments affect attitudes are likely applicable across various groups, the absolute strength of arguments may not generalize fully beyond the student demographic used in this study.

Third, although we demonstrated the outcomes of the mutual presentation of strong weak arguments, we did not explicitly investigate the mechanisms that explain these effects. Hence, future research could examine individuals’ thought processes when they are presented with distinctly weak, moderate, and strong arguments.

Returning to the opening scenario, what can we recommend to someone who wants to convince others to live a healthier lifestyle, practice sustainable behavior, or support a certain political agenda? If you have strong arguments, you should certainly mention all of them. If you can think of arguments of moderate strength, go ahead and include them to make your argument even more convincing. If you have only come up with weak arguments to supplement the strong arguments, however, it would be better to keep them to yourself to avoid weakening your argumentation. In this case, it is actually true that less is more.

## Data Availability

The data that support the findings of this study are available from the corresponding author upon reasonable request.

## References

[1] Hovland, C. I., Janis, I. L. & Kelley, H. H. *Communication and Persuasion. Psychological Studies of Opinion Change* (Yale University Press, 1953).

[2] Sikkink, D. E. An experimental study of the effects on the listener of anticlimax order and authority in an argumentative speech. *South. Speech J.* **22**, 73–78 (1956).

[3] O’Keefe, D. J. How to handle opposing arguments in persuasive messages: A meta-analytic review of the effects of one-sided and two-sided messages. *Ann. Int. Commun. Assoc.* **22**, 209–249 (1999).

[4] Gilkinson, H., Paulson, S. F. & Sikkink, D. E. Effects of order and authority in an argumentative speech. *Q. J. Speech* **40**, 183–192 (1954).

[5] Sponberg, H. A study of the relative effectiveness of climax and anti-climax order in an argumentative speech. *Speech Monogr.* **13**, 35–44 (1946).

[6] Helweg-Larsen, M. & Howell, C. Effects of erotophobia on the persuasiveness of condom advertisements containing strong or weak arguments. *Basic Appl. Soc. Psychol.* **22**, 111–117 (2000).

[7] Weaver, K., Hock, S. J. & Garcia, S. M. Top 10 reasons: When adding persuasive arguments reduces persuasion. *Mark. Lett.* **27**, 27–38 (2016).

[8] Nisbett, R. E., Zukier, H. & Lemley, R. E. The dilution effect: Nondiagnostic information weakens the implications of diagnostic information. *Cognit. Psychol.* **13**, 248–277 (1981).

[9] Yadav, M. S. How buyers evaluate product bundles: A model of anchoring and adjustment. *J. Consum. Res.* **21**, 342–353 (1994).

[10] Weaver, K., Garcia, S. M. & Schwarz, N. The presenter’s paradox. *J. Consum. Res.* **39**, 445–460 (2012).

[11] Friedrich, J., Fetherstonhaugh, D., Casey, S. & Gallagher, D. Argument integration and attitude change: Suppression effects in the integration of one-sided arguments that vary in persuasiveness. *Pers. Soc. Psychol. Bull.* **22**, 179–191 (1996).

[12] Nisbett, R. E., Peng, K., Choi, I. & Norenzayan, A. Culture and systems of thought: Holistic versus analytic cognition. *Psychol. Rev.* **108**, 291–310 (2001). 11381831 10.1037/0033-295x.108.2.291

[13] Angeles, P. A. *Dictionary of Philosophy* (Barnes & Noble, 1981).

[14] Hoeken, H., Hornikx, J. & Linders, Y. The importance and use of normative criteria to manipulate argument quality. *J. Advert.* **49**(2), 195–201 (2020).

[15] Jordan, J. Pragmatic arguments and belief. *Am. Philos. Q.* **33** (4), 409–420 (1996).

[16] Walton, D. *Argumentation Schemes for Presumptive Reasoning* (Erlbaum, 1996).

[17] Anderson, N. H. Averaging versus adding as a stimulus-combination rule in impression formation. *J. Exp. Psychol.* **70**, 394–400 (1965). 5826027 10.1037/h0022280

[18] Hogarth, R. M. & Einhorn, H. J. Order effects in belief updating: The belief-adjustment model. *Cognit. Psychol.* **24**, 1–55 (1992).

[19] Gaeth, G. J., Levin, I. P., Chakraborty, G. & Levin, A. M. Consumer evaluation of multi-product bundles: an information integration analysis. *Mark. Lett.* **2**, 47–58 (1990).

[20] Petty, R. E. & Cacioppo, J. T. The effects of involvement on responses to argument quantity and quality: Central and peripheral routes to persuasion. *J. Personal. Soc. Psychol.* **46**, 69–81 (1984).

[21] Meyvis, T. & Janiszewski, C. Consumers’ beliefs about product benefits: The effect of obviously irrelevant information. *J. Consum. Res.* **28**, 618–636 (2002).

[22] Anderson, N. H. Integration theory and attitude change. *Psychol. Rev.* **78**, 171–206 (1971).

[23] Shanteau, J. Averaging versus multiplying combination rules of inference judgment. *Acta. Psychol.* **39**, 83–89 (1975).

[24] Corneille, O. & Judd, C. M. Accentuation and sensitization effects in the categorization of multifaceted stimuli. *J. Personal. Soc. Psychol.* **77**, 927–941 (1999). 10.1037//0022-3514.77.5.927 10573873

[25] Ilicic, J. & Webster, C. M. Celebrity co-branding partners as irrelevant brand information in advertisements. *J. Bus. Res.* **66**, 941–947 (2011).

[26] Tetlock, P. E., Lerner, J. S. & Boettger, R. The dilution effect: Judgmental bias, conversational convention, or a bit of both?. *Eur. J. Social Psychol.* **26**, 915–934 (1996).

[27] Gierl, H. & Großmann, T. Imply-Benefit-Attribute Im Bereich häufig gekaufter Konsumgüter. *Schmalenbachs Z. für Betriebswirtschaftliche Forschung* **60**, 355–384 (2008).

[28] Troutman, C. M. & Shanteau, J. Do consumers evaluate products by adding or averaging attribute information?. *J. Consum. Res.* **3**, 101–106 (1976).

[29] Petty, R. E. & Wegener, D. T. Thought systems, argument quality, and persuasion. In *The Content, Structure, and Operation of Thought Systems* (eds Wyer, R. S., Jr. & Srull, T. K.) 147–162 (Erlbaum, 1991).

[30] SoSci Panel (2024). https://www.soscipanel.de/index.php

[31] Insko, C. A. One-sided versus two-sided communications and countercommunications. *J. Abnorm. Soc. Psychol.* **65**, 203–206 (1962).

[32] Carpenter, C. J. A. meta-analysis of the ELM’s argument quality × processing type predictions. *Hum. Commun. Res.* **41**, 501–534 (2015).

[33] Walton, D. N. What is reasoning? What is an argument? *J. Philos.* **87** (8), 399–419 (1990).

[34] Koch, T. & Zerback, T. Helpful or harmful? How frequent repetition affects perceived statement credibility. *J. Commun.* **63**, 993–1010 (2013).

[35] Akhtar, O., Paunesku, D. & Tormala, Z. L. Weak > strong: The ironic effect of argument strength on supportive advocacy. *Pers. Soc. Psychol. Bull.* **39**, 1214–1226 (2013). 23798375 10.1177/0146167213492430

